# Does the evolution of micromorphology accompany chromosomal changes on dysploid and polyploid levels in the *Barnardia japonica* complex (Hyacinthaceae)?

**DOI:** 10.1186/s12870-023-04456-9

**Published:** 2023-10-11

**Authors:** Hyeonjin Kim, Bokyung Choi, Changyoung Lee, Jin-Hyub Paik, Chang-Gee Jang, Hanna Weiss-Schneeweiss, Tae-Soo Jang

**Affiliations:** 1 Department of Biological Science, College of Bioscience and Biotechnology, Chungnam National University, Daejeon, Republic of Korea; 2 International Biological Material Research Center, Korea Research Institute of Bioscience and Biotechnology, Daejeon, Republic of Korea; 3 Department of Biology Education, Kongju National University, Gongju, 32588 Republic of Korea; 4 Department of Botany and Biodiversity Research, University of Vienna, Vienna, A-1030 Austria

**Keywords:** *Barnardia japonica* complex, Cytotype variation, Genome downsizing, Hybridization, Polyploidy

## Abstract

**Background:**

Chromosome number and genome size changes via dysploidy and polyploidy accompany plant diversification and speciation. Such changes often impact also morphological characters. An excellent system to address the questions of how extensive and structured chromosomal changes within one species complex affect the phenotype is the monocot species complex of *Barnardia japonica*. This taxon contains two well established and distinct diploid cytotypes differing in base chromosome numbers (AA: *x* = 8, BB: *x* = 9) and their allopolyploid derivatives on several ploidy levels (from 3*x* to 6*x*). This extensive and structured genomic variation, however, is not mirrored by gross morphological differentiation.

**Results:**

The current study aims to analyze the correlations between the changes of chromosome numbers and genome sizes with palynological and leaf micromorphological characters in diploids and selected allopolyploids of the *B. japonica* complex. The chromosome numbers varied from 2*n* = 16 and 18 (2*n* = 25 with the presence of supernumerary B chromosomes), and from 2*n* = 26 to 51 in polyploids on four different ploidy levels (3*x*, 4*x*, 5*x*, and 6*x*). Despite additive chromosome numbers compared to diploid parental cytotypes, all polyploid cytotypes have experienced genome downsizing. Analyses of leaf micromorphological characters did not reveal any diagnostic traits that could be specifically assigned to individual cytotypes. The variation of pollen grain sizes correlated positively with ploidy levels.

**Conclusions:**

This study clearly demonstrates that karyotype and genome size differentiation does not have to be correlated with morphological differentiation of cytotypes.

**Supplementary Information:**

The online version contains supplementary material available at 10.1186/s12870-023-04456-9.

## Introduction

Chromosome number changes comprising polyploidy, and dysploidy, play an important role in plant genome diversification and have thus been frequently analyzed to better understand the angiosperm evolution [1, 2]. Polyploidy (i.e., the whole-genome duplication) including both auto- and allopolyploidy is one of the major processes involved in the diversification and speciation of plants [2–6]. Autopolyploidy has received relatively little attention due to high levels of morphological similarity between autopolyploids and their parental diploid taxa [7–9]. In contrast, allopolyploids have been studied extensively in several plant groups with focus on changes of genome sizes and karyotypes compared to their parental taxa (e.g., *Brassica* L. [10]; *Melampodium* L. [11, 12]; *Nicotiana* L. [13]; *Prospero* Salisb. [14]; *Tragopogon* L. [15]). Although polyploidy is recognized as process leading to plant diversification in natural populations, comparative analyses of the evolution of polyploid genomes are rather scarce and limited to several plant groups [11, 12, 14, 16–18].

Two mechanisms result in the stepwise change of the haploid chromosome number among related species [1, 19], aneuploidy and dysploidy. While aneuploidy is less relevant to diversification and evolution as it introduces immediate genetic imbalance by addition or removal of single chromosomes, dysploidy contributes to evolution by changing haploid chromosome numbers via chromosomal rearrangements and keeping changes of DNA amount to minimum [1, 2]. Dysploidy and auto- and allopolyploidy, with occasional indication of aneuploidy on higher ploidy levels within species complexes have been reported in many species in family Hyacinthaceae Batsch ex Borkh. (also treated as Asparagaceae Juss.; [14, 20–23]). The genus *Barnardia* Lindl. comprises two geographically disjunct species groups: *B. numidica* (Poir.) Speta and the species complex referred to as *B. japonica* (Thunb.) Schult. & Schult.f. [24–26]. *Barnardia numidica* is widespread in the Balearic Islands and North-West Africa (Algeria, Tunisia, and Libya) and is known only as diploid with 2*n* = 18 (*x* = 9; [27]). In contrast, the *B. japonica* complex is found in Eastern Asia and exhibits a spectacular array of chromosomal variation with two different base chromosome numbers (*x* = 8 and 9), an extensive range of polyploids (3*x*, 4*x*, 5*x* and 6*x*), various types of chromosomal polymorphisms including the presence of B-chromosomes [28, 29], as well as genome size variation [22]. Molecular phylogenetic studies of plastid and nuclear DNA sequence data have revealed that the *B. japonica* complex is the most basal clade in the family of Hyacinthaceae [30, 31].

Two distinct and stable diploid cytotypes with base chromosome numbers of either *x* = 8 (cytotype/genome A) and *x* = 9 (cytotype/genome B) have been described in the *B. japonica* complex [25, 28, 32]. Diploids of AA (2*n* = 2*x* = 16) and BB (2*n* = 2*x* = 18) cytotypes are known to hybridize and to form allopolyploids in natural populations. Diploid homoploid hybrids AB (2*n* = 2*x* = 17), as well as a myriad of polyploids of various genomic compositions have been reported (ABB, 2*n* = 3*x* = 26; BBB, 2*n* = 3*x* = 27; AAAA, 2*n* = 4*x* = 32; AABB, 2*n* = 4*x* = 34; ABBB, 2*n* = 4*x* = 35; BBBB, 2*n* = 4*x* = 36; AABBB, 2*n* = 5*x* = 43; AAABBB, 2*n* = 6*x* = 51; [24, 25, 33, 34]). The diploid AA cytotype is found in China and Korea covering nearly the entire distribution range of the *B. japonica* complex, with the exception of Japan [24, 25]. In contrast, the BB cytotype is geographically more restricted to a narrow area of eastern central China, Jeju Island in Korea, and Japan [35]. It is commonly accepted to refer to the hybrids between different, karyotypically well differentiated cytotypes (particularly in monocots), as allopolyploids, despite the fact that the diploid cytotypes are all part of the same species complex. We are, therefore, going to use the same approach. Allotetraploids of genomic constitution AABB are frequently found throughout the complex distribution range [25, 33, 36]. Cytogenomic analyses of AABB allotetraploids revealed that the parental subgenomes are retained without any rearrangements except for the loss of NOR (e.g., nucleolar organizer region) in the parental A-genome [25, 37]. Supernumerary genetic material, either as B-chromosomes (Bs) or supernumerary chromosomal segments (SCSs) that are physically integrated into the standard chromosome complement, is frequently found in various plant groups, particularly in monocots, with hot spots in Liliales and Commelinales [38–43]. The SCSs can be located either interstitially or terminally and easily identified in homologous chromosomes in the heterozygous condition due to chromosome length differences [2, 14]. Both SCSs and Bs are often, but not always, heterochromatic. B chromosomes are also dispensable components of the genomes and usually behave as selfish genetic elements [2, 40]. In plants, frequency of both SCSs and Bs in diploids and polyploids are similar [44, 45] but tends to be higher in taxa with large genome sizes [39, 46]. Although the presence of Bs have been well documented in the *B. japonica* complex [28, 29, 33], the occurrence of SCSs has yet to be shown [29, 33].

The plants of the genus *Barnardia* possess rather large bulbs (over 4 cm in diameter) with several brown scales, an elongated raceme with 30–70 purple flowers, white or pale pink tepals including six stamens, and a central pistil [27, 31, 47]. The micromorphology of leaf epidermis and characters of pollen grains are often used as a diagnostic character to establish relationships and to define major groups in family Hyacinthaceae [48–50] as well as other economically important plant families such as Lamiaceae Martinov [51], Polygonaceae Juss. [52], Melastomataceae Juss. [53], Asteraceae Giseke [54], Poaceae Barnhart [55], and Iridaceae Juss. [56–58]. Specifically, wax striation and size of stomatal complexes are considered to be important diagnostic value in Hyacinthaceae [48]. Few studies to date have addressed the question of evolution of those characters in relations to extensive dysploidy and polyploidy within species complexes. Similarly, no morphological diagnostic characters have so far been found to allow for identification of the two diploid cytotypes and their hybrid/polyploid descendants in the *B. japonica* complex [25]. Thus, the current species concept in the *B. japonica* complex is mostly based on classical karyotaxonomic studies with two diploid cytotypes differing in base chromosome number and a myriad of resulting polyploid cytotypes [22, 24–26, 28, 35, 59], while the underlying patterns of genetic and micromorphological differentiation are still unknown.

Thus, the main goals of this study are (1) to establish chromosome numbers, karyotype structures, and genome sizes of 131 diploid and polyploid individuals of the *B. japonica* complex collected from natural populations in South Korea; (2) to analyze the leaf and pollen micromorphology in diploid and polyploid cytotypes of various genomic constitution, and (3) to correlate the variation of the analyzed characters with structured karyotypic variation in an attempt to identify new diagnostic characters or character combinations for cytotype identification in the *Barnardia japonica* complex.

## Materials and methods

### Sampling, chromosome counts, and karyotype analysis

**Fig. 1:**
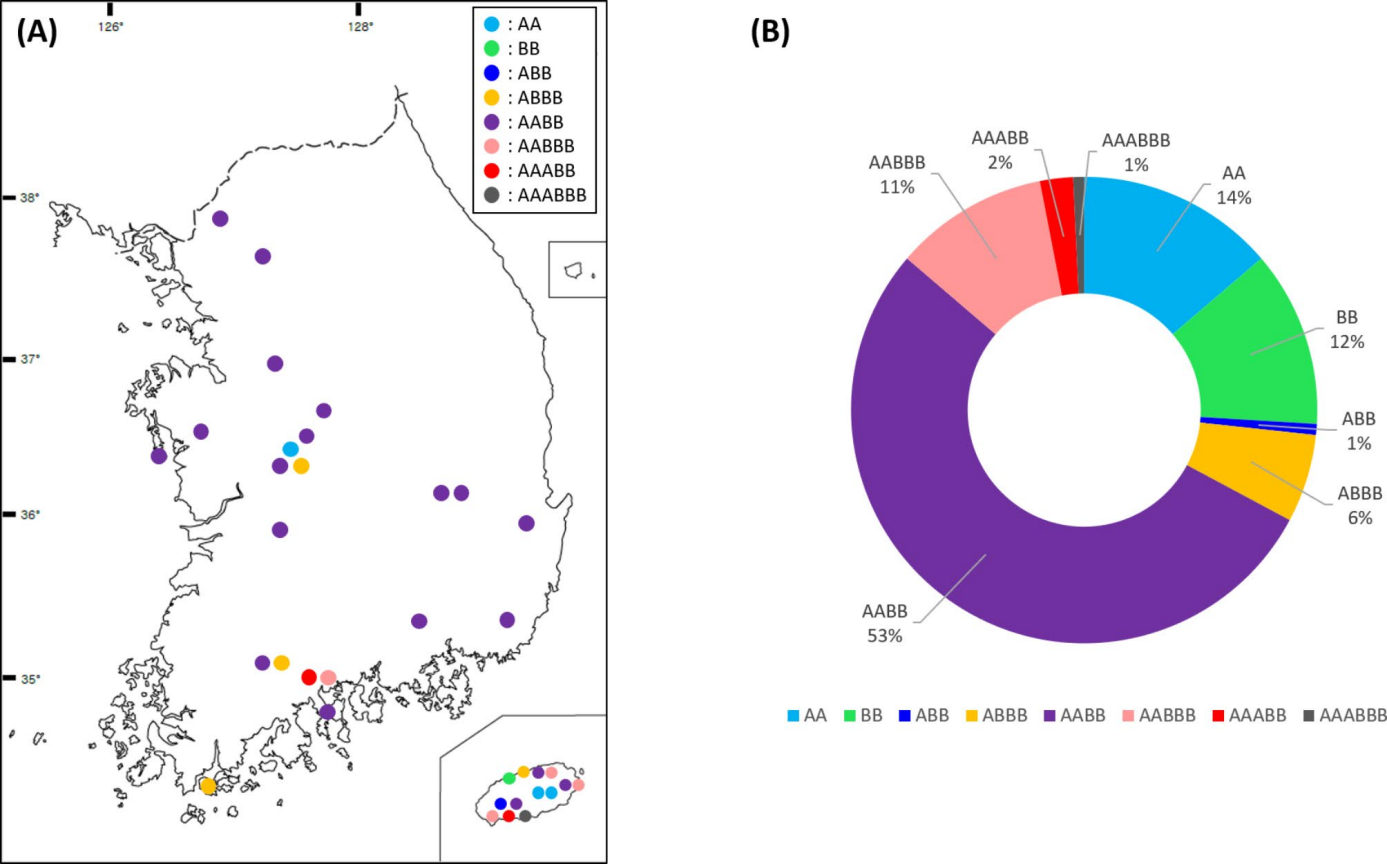
Populations of the *Barnardia japonica* complex analysed in the current study (Table 1). (**A**) Map with the locations of sampled diploid and polyploid populations; (**B**) Proportions of diploid and polyploid cytotypes among investigated 131 individuals

Bulbs of the *Barnardia japonica* complex were collected in the field between 2017 and 2022 in South Korea (Fig. 1) and cultivated at Chungnam National University. Sampling of diploid and polyploid cytotypes was guided by earlier studies [25] with the aim to collect most of the ploidy levels reported. Thirty-four diploid individuals representing two genomes differing in base chromosome numbers (A genome: *x* = 8 and B genome: *x* = 9) and 97 polyploid individuals representing four ploidy levels (3*x*, 4*x*, 5*x* and 6*x*) were collected from 34 natural populations (Table 1).

**Table 1:** Individuals of the *Barnardia japonica* complex analyzed in this study with detailed voucher information

Cytotype	Collection number	Locality; GPS coordinate	2*n*	Genome size (1 C value)
AA	SD01-7^3^	Sinseong-dong, Daejeon; N 36° 23’ 07” E 127° 20’ 37”, 97 m	16	12.46
	SD01-9	Sinseong-dong, Daejeon; N 36° 23’ 07” E 127° 20’ 37”, 97 m	16	12.38
	SD01-10	Sinseong-dong, Daejeon; N 36° 23’ 07” E 127° 20’ 37”, 97 m	16	11.87
	SD01-13	Sinseong-dong, Daejeon; N 36° 23’ 07” E 127° 20’ 37”, 97 m	16	-
	JCKC190422^1^	Mojioreum, Jeju-do; N 33° 23’ 40” E 126° 45’ 59”, 296 m	16	-
	JCKC190474	Mojioreum, Jeju-do; N 33° 23’ 41” E 126° 45’ 58”, 252 m	16	-
	JCKC190476^1,3^	Mojioreum, Jeju-do; N 33° 23’ 41” E 126° 45’ 58”, 252 m	16	-
	JSH01-1^1^	Mojioreum, Jeju-do; N 33° 23’ 43” E 126° 46’ 00”, 388 m	16	-
	JSH01-2	Mojioreum, Jeju-do; N 33° 23’ 43” E 126° 46’ 00”, 388 m	16	-
	JSH01-3^2^	Mojioreum, Jeju-do; N 33° 23’ 43” E 126° 46’ 00”, 388 m	16	-
	JSH01-7^2^	Mojioreum, Jeju-do; N 33° 23’ 43” E 126° 46’ 00”, 388 m	16	-
AA + 1B	JCKC190423	Mojioreum, Jeju-do; N 33° 23’ 40” E 126° 45’ 59”, 296 m	19	-
	JCKC190475^3^	Mojioreum, Jeju-do; N 33° 23’ 41” E 126° 45’ 58”, 252 m	19	-
	JSH01-5	Mojioreum, Jeju-do; N 33° 23’ 43” E 126° 46’ 00”, 388 m	19	-
AA + 2Bs	HH01-6^1,3^	Mojioreum, Jeju-do; N 33° 23’ 24” E 126° 46’ 12”, 293 m	20	-
AA + 3Bs	JSH01-10	Mojioreum, Jeju-do; N 33° 23’ 43” E 126° 46’ 00”, 388 m	21	-
AA + 4Bs	JSH01-4	Mojioreum, Jeju-do; N 33° 23’ 43” E 126° 46’ 00”, 388 m	22	-
AA + 5Bs	HALLA47	Mt. Yeongju, JeJu-do; N 33° 24’ 00” E 126° 47’ 59”, 229 m	23	12.28
BB	JH01-1^2,3^	Dojeogul, Jeju-do; N 33° 29’ 49” E 126° 29’ 37”, 178 m	18	-
	JH01-3^1^	Dojeogul, Jeju-do; N 33° 29’ 49” E 126° 29’ 37”, 178 m	18	-
	JE02-1	Dojeogul, Jeju-do; N 33° 29’ 50” E 126° 29’ 38”, 90 m	18	9.05
	JE02-4^2,3^	Dojeogul, Jeju-do; N 33° 29’ 50” E 126° 29’ 38”, 90 m	18	-
	DG15	Dojeogul, Jeju-do; N 33° 29’ 52” E 126° 29’ 36”, 61 m	18	9.18
	DG16	Dojeogul, Jeju-do; N 33° 29’ 52” E 126° 29’ 36”, 61 m	18	9.28
BB + 1B	JH01-2^1,3^	Dojeogul, Jeju-do; N 33° 29’ 49” E 126° 29’ 37”, 178 m	19	-
	JE02-16	Dojeogul, Jeju-do; N 33° 29’ 50” E 126° 29’ 38”, 90 m	19	-
	DG03	Dojeogul, Jeju-do; N 33° 29’ 52” E 126° 29’ 36”, 61 m	19	9.30
BB + 2Bs	JE02-17	Dojeogul, Jeju-do; N 33° 29’ 50” E 126° 29’ 38”, 90 m	20	-
BB + 3Bs	JE02-3	Dojeogul, Jeju-do; N 33° 29’ 50” E 126° 29’ 38”, 90 m	21	-
	JE02-5	Dojeogul, Jeju-do; N 33° 29’ 50” E 126° 29’ 38”, 90 m	21	9.52
	JE02-6	Dojeogul, Jeju-do; N 33° 29’ 50” E 126° 29’ 38”, 90 m	21	9.13
	JE02-15	Dojeogul, Jeju-do; N 33° 29’ 50” E 126° 29’ 38”, 90 m	21	9.44
BB + 5Bs	JE02-8	Dojeogul, Jeju-do; N 33° 29’ 50” E 126° 29’ 38”, 90 m	23	-
BB + 7Bs	JE02-18	Dojeogul, Jeju-do; N 33° 29’ 50” E 126° 29’ 38”, 90 m	25	-
ABB	JEJU037^1,2,3^	Sangmo-ri, Jeju-do; N 33° 12’ 03” E 126° 16’ 20”, 14 m	26	-
ABBB	WD59	Wan-do, Jeonnam; N 34° 19’ 34” E 126° 51’ 11”, 20 m	35	18.79
	JHMM12-6^3^	Suncheon, Jeonnam; N 35° 04’ 08” E 127° 27’ 25”, 218 m	35	18.90
ABBB + 1B	WD54	Wan-do, Jeonnam; N 34° 19’ 34” E 126° 51’ 11”, 20 m	36	19.02
	DG27	Dodubong park, Jeju-do; N 33° 30’ 29” E 126° 28’ 06”, 39 m	36	-
ABBB + 2Bs	WD60	Wan-do, Jeonnam; N 34° 19’ 34” E 126° 51’ 11”, 20 m	37	18.67
ABBB + 3Bs	HY2018-003^1^	Gung-dong, Daejeon; N 36° 22’ 17” E 127° 20’ 34”, 60 m	38	-
	HY2018-026	Gung-dong, Daejeon; N 36° 22’ 17” E 127° 20’ 34”, 60 m	38	-
	JCKC190429	Albamoreum, Jeju-do; N 33° 29’ 15” E 126° 42’ 27”, 252 m	38	-
AABB	HH01-1	Mt. Cheonma, Gyeonggi-do; N 37° 41’ 24” E 127° 24’ 36”, 157 m	34	-
	J01-9^3^	Munsan, Gyeonggi-do; N 37° 51’ 00” E 126° 46’ 00”; 9 m	34	-
	KHJ06	Jeungpyeong, Chungbuk; N 36° 45’ 00” E 127° 36’ 27”, 109 m	34	-
	CJ01-8	Mt. Yangseong, Chungbuk; N 36° 30’ 30” E 127° 29’ 11”, 198 m	34	20.26
	CJ01-14	Mt. Jakdu, Chungbuk; N 36° 30’ 47” E 127° 29’ 21”, 201 m	34	19.80
	SCK00024	Mt. Bonghwa, Chungnam; N 36° 47’ 06” E 126° 26’ 22”, 76 m	34	-
	SCK00025	Mt. Bonghwa, Chungnam; N 36° 47’ 06” E 126° 26’ 22”, 76 m	34	-
	SCK00026	Mt. Bonghwa, Chungnam; N 36° 47’ 06” E 126° 26’ 22”, 76 m	34	-
	NOK210514-28	Nok-do, Chungnam; N 36° 16’ 37” E 126° 16’ 04”, 49 m	34	-
	NOK210514-30	Nok-do, Chungnam; N 36° 16’ 37” E 126° 16’ 04”, 49 m	34	-
	NOK210514-34	Nok-do, Chungnam; N 36° 16’ 37” E 126° 16’ 04”, 49 m	34	-
	HY2018-005	Gung-dong, Daejeon; N 36° 22’ 13” E 127° 20’ 34”, 75 m	34	-
	HY2018-006	Gung-dong, Daejeon; N 36° 22’ 13” E 127° 20’ 34”, 75 m	34	-
	HY2018-007	Gung-dong, Daejeon; N 36° 22’ 13” E 127° 20’ 34”, 75 m	34	-
	HY2018-008^1^	Gung-dong, Daejeon; N 36° 22’ 13” E 127° 20’ 34”, 75 m	34	-
	HY2018-014^3^	Gung-dong, Daejeon; N 36° 22’ 28” E 127° 20’ 38”, 67 m	34	-
	HY2018-016	Gung-dong, Daejeon; N 36° 22’ 28” E 127° 20’ 38”, 67 m	34	-
	HY2018-018	Gung-dong, Daejeon; N 36° 22’ 28” E 127° 20’ 38”, 67 m	34	-
	HY2018-019	Gung-dong, Daejeon; N 36° 22’ 28” E 127° 20’ 38”, 67 m	34	-
	MHS03-2	Gung-dong, Daejeon; N 36° 22’ 28” E 127° 20’ 38”, 67 m	34	-
	MHS03-3	Gung-dong, Daejeon; N 36° 22’ 28” E 127° 20’ 38”, 67 m	34	19.69
	MHS03-4	Gung-dong, Daejeon; N 36° 22’ 28” E 127° 20’ 38”, 67 m	34	-
	MHS05-3	Gung-dong, Daejeon; N 36° 22’ 28” E 127° 20’ 38”, 67 m	34	20.23
	MHS05-4	Gung-dong, Daejeon; N 36° 22’ 28” E 127° 20’ 38”, 67 m	34	-
	HA01-11	Wanju, Jeonbuk; N 36° 03’ 41” E 127° 17’ 19”, 269 m	34	-
	JHMM11-10	Mt. Jogye, Jeonnam; N 34° 59’ 16” E 127° 17’ 13”, 479 m	34	19.77
	BD3	Baek-do, Jeonnam; N 34° 03’ 19” E 127° 35’ 03”, 54 m	34	-
	JCKC1834	Gasan, Gyeongbuk; N 36° 02’ 45” E 128° 33’ 56”, 631 m	34	-
	HA10-2	Haman, Gyeongnam; N 35° 15’ 55” E 128° 24’ 30”, 64 m	34	19.95
	J01-4	Mt. Pal-Gong, Daegu; N 36° 01’ 00” E 128° 35’ 00”; 282 m	34	-
	C01-1^3^	Mt. Geumjeong, Busan; N 35° 16’ 00” E 129° 01’ 59”; 361 m	34	-
	J10-7	Mt. Geumjeong, Busan; N 35° 17’ 36” E 129° 01’ 27”, 33 m	34	-
	J10-9	Mt. Geumjeong, Busan; N 35° 17’ 36” E 129° 01’ 27”, 33 m	34	-
	JE01-7	Yeon-dong, Jeju-do; N 33° 29’ 28” E 126° 29’ 25”, 96 m	34	19.22
	JE01-8	Yeon-dong, Jeju-do; N 33° 29’ 28” E 126° 29’ 25”, 96 m	34	-
	JE04-10	Yongdam-dong, Jeju-do; N 33° 31’ 12” E 126° 29’ 52”, 7 m	34	-
	JCKC190443	Geomioreum, Jeju-do; N 33° 26’ 53” E 126° 48’ 01”, 297 m	34	-
	JCKC190478^2^	Mojioreum, Jeju-do; N 33° 23’ 58” E 126° 46’ 11”, 170 m	34	-
	JJ11-7	Sangmo-ri, Jeju-do; N 33° 11’ 58” E 126° 17’ 26”, 69 m	34	16.80
	JEJU038	Sangmo-ri, Jeju-do; N 33° 12’ 03” E 126° 16’ 20”, 14 m	34	-
	JEJU040^1,2,3^	Sangmo-ri, Jeju-do; N 33° 12’ 03” E 126° 16’ 20”, 14 m	34	-
	JEJU054	Sangmori, Jeju-do; N 33° 11’ 56” E 126° 16’ 24”, 5 m	34	17.91
	JEJU055	Sangmori, Jeju-do; N 33° 11’ 56” E 126° 16’ 24”, 5 m	34	17.70
	JEJU058	Sangmori, Jeju-do; N 33° 11’ 56” E 126° 16’ 24”, 5 m	34	17.50
	HALLA74	Seopjikoji, Jeju-do; N 33° 25’ 44” E 126° 55’ 46”, 98 m	34	20.18
	HALLA75	Seopjikoji, Jeju-do; N 33° 25’ 44” E 126° 55’ 46”, 98 m	34	19.72
	JJ20-2	Seosaroreum, Jeju-do; N 33° 12’ 20” E 126° 16’ 54”, 28 m	34	17.05
	JJ20-5	Seosaroreum, Jeju-do; N 33° 12’ 20” E 126° 16’ 54”, 28 m	34	17.24
	JJ20-15	Seosaroreum, Jeju-do; N 33° 12’ 20” E 126° 16’ 54”, 28 m	34	-
	JJ11-2	Seosaroreum, Jeju-do; N 33° 12’ 20” E 126° 16’ 52”, 26 m	34	17.67
	JJD01	Sagye Beach, Jeju-do; N 33° 13’ 27” E 126° 17’ 52”, 6 m	34	18.51
AABB + 1B	HH01-2	Mt. Cheonma, Gyeonggi-do; N 37° 41’ 24” E 127° 24’ 36”, 157 m	35	-
	HH01-4	Mt. Cheonma, Gyeonggi-do; N 37° 41’ 24” E 127° 24’ 36”, 157 m	35	-
	J01-8	Munsan-eup, Gyeonggi-do; N 37° 51’ 00” E 126° 46’ 00”, 9 m	35	-
	KHJ04	Jeungpyeong, Chungbuk; N 36° 45’ 00” E 127° 36’ 27”, 109 m	35	-
	JCKC190479^3^	Mojioreum, Jeju-do; N 33° 23’ 58” E 126° 46’ 11”, 170 m	34	-
	BD4	Baek-do, Jeonnam; N 34° 03’ 19” E 127° 35’ 03”, 54 m	35	20.32
AABB + 2Bs	J01-5	Munsan-eup, Gyeonggi-do; N 37° 51’ 00” E 126° 46’ 00”, 9 m	36	20.19
	J01-6	Munsan-eup, Gyeonggi-do; N 37° 51’ 00” E 126° 46’ 00”, 9 m	36	-
	KHJ13-2	Jeungpyeon, Chungbuk; N 36° 45’ 9” E 127° 36’ 26”, 101 m	36	-
	Gasan20-40	Mt. Ga, Gyeongbuk; N 36° 02’ 25” E 128° 34’ 19”, 783 m	36	20.03
	JCKC190410	Siksan-bong, Jeju-do; N 33° 27’ 54” E 126° 55’ 07”, 3 m	36	-
	JCKC190429	Albamoreum, Jeju-do; N 33° 29’ 15” E 126° 42’ 27”, 252 m	36	-
AABB + 5Bs	HH01-5	Mt. Cheonma, Gyeonggi-do; N 37° 41’ 24” E 127° 24’ 36”, 157 m	39	-
	KHJ07	Jeungpyeong, Chungbuk; N 36° 45’ 00” E 127° 36’ 27”, 109 m	39	-
	BKC925	Gyeongju, Gyeongbuk; N 35° 48’ 24” E 129° 05’ 49”, 486 m	39	-
	J01-13^3^	Yeosu, Jeonnam; N 34° 53’ 49” E 127° 42’ 41”, 16 m	39^*^	-
AABB + 6Bs	KHJ05^3^	Jeungpyeong, Chungbuk; N 36° 45’ 00” E 127° 36’ 27”, 109 m	40	-
	HK03	Cheongwon, Chungbuk; N 36° 36’ 24” E 127° 34’ 49”, 364 m	40	21.38
	BKC923	Gyeongju, Gyeongbuk; N 35° 48’ 24” E 129° 05’ 49”, 486 m	40	-
AABBB	J01-16^3^	Yeosu, Jeonnam; N 34° 53’ 49” E 127° 42’ 41”, 16 m	43^*^	-
	J01-17^1,3^	Yeosu, Jeonnam; N 34° 53’ 49” E 127° 42’ 41”, 16 m	43^*^	-
	JEJU039^2,3^	Sangmo-ri, Jeju-do; N 33° 12’ 03” E 126° 16’ 20”, 14 m	43	-
	JCKC190483	Siksanbong, Jeju-do; N 33° 27’ 57” E 126° 55’ 12”, 13 m	43	-
	JCKC190484	Siksanbong, Jeju-do; N 33° 27’ 57” E 126° 55’ 12”, 13 m	43	-
	JCKC190491	Siksanbong, Jeju-do; N 33° 27’ 57” E 126° 55’ 12”, 13 m	43	22.03
	HALLA85	Seongsan-eup, Jeju-do; N 33° 25’ 44” E 126° 55’ 46”, 98 m	43	22.36
	HALLA89	Seongsan-eup, Jeju-do; N 33° 25’ 44” E 126° 55’ 46”, 98 m	43	22.95
	DG19	Dodubong park, Jeju-do; N 33° 30’ 29” E 126° 28’ 06”, 39 m	43	22.89
AABBB + 1B	J01-12	Yeosu, Jeonnam; N 34° 53’ 49” E 127° 42’ 41”, 16 m	44	-
	JCKC190431	Mojioreum, Jeju-do; N 33° 26’ 51” E 126° 47’ 57”, 267 m	44	-
AABBB + 2Bs	JCKC190481	Siksanbong, Jeju-do; N 33° 27’ 57” E 126° 55’ 12”, 13 m	45	-
AABBB + 3Bs	WD21	Wan-do, Jeonnam; N 34° 19’ 33” E 126° 51’ 11”, 0 m	46	23.23
	JCKC190439	Geomioreum Jeju-do; N 33° 27’ 07” E 126° 47’ 55”, 254 m	46	22.42
AAABB	J01-10^1,3^	Yeosu, Jeonnam; N 34° 53’ 49” E 127° 42’ 41”, 16 m	42	-
AAABB + 6Bs	J01-11	Yeosu, Jeonnam; N 34° 53’ 49” E 127° 42’ 41”, 16 m	44^*, **^	-
	J01-15^3^	Yeosu, Jeonnam; N 34° 53’ 49” E 127° 42’ 41”, 16 m	44^*, **^	-
AAABBB	JEJU041^1,2,3^	Sangmo-ri, Jeju-do; N 33° 12’ 03” E 126° 16’ 20”, 14 m	51	-

Note: Superscript of collection numbers indicated the leaf (^1^), pollen micromorphological (^2^) and karyotype analysis (^3^), respectively. ^*^: individual possessing supernumerary chromosomal segments. ^**^: Aneuploidy

Chromosome numbers and karyotypes were determined using standard Feulgen staining [60]. In total, chromosome numbers of 131 collected plants of the *B. japonica* complex were established. Twenty-two plants of representing all cytotype were karyotyped to aid identification of their genomic composition ( [25, 28, 34]; Table 1 and Table S1). For cytological investigations, actively growing root meristems were pretreated with 0.05% aqueous solution of colchicine for 4 h at room temperature, fixed in ethanol : acetic acid (3:1), and stored at − 20℃ until use. Root tips were hydrolyzed in 5 N HCl (VWR; Vienna, Austria) at room temperature for 30 min and stained with Schiff’s reagent (Sigma, Vienna, Austria) for 60 min in darkness. Squash preparations were made in a drop of 60% acetic acid. At least three well-spread and complete metaphase plates with a medium degree of chromosome condensation were chosen for karyotyping for each individual. Chromosomes were cut out and karyotypes arranged using Adobe Photoshop CS6. Chromosomes were measured using Micromeasure ver. 3.3 (< www.colostate.edu/Depts/Biology/MicroMeasure/>) following [61]. Chromosome arm lengths and total chromosome lengths were measured in at least three chromosomal spreads per individual (unless otherwise indicated; Table S1).

### Genome size measurements

Genome sizes of 42 selected individuals of the *B. japonica* complex were measured using flow cytometry with *Pisum sativum* “Kleine Rheinländerin” (4.42 pg/1 C) for diploids and *Vicia faba* L. “Inovec” (13.45 pg/1 C) for polyploids as internal standards [62–65]. The methodology used for the measurement of the genome sizes followed [64, 66]. Measurements were performed using Sysmex CyFlow Cytometer (Sysmex Partec GmbH, Görlitz, Germany) and 1 C values were calculated following [63]. The CVs (coefficient values) of all measurements were usually lower than 3% and never exceeded 5% [66]. To test whether the genome sizes of each cytotype with and without B chromosomes was significantly different, the Mann-Whitney test was performed in GraphPad Prism version 9.

### Leaf and pollen micromorphological traits

Fresh leaf and pollen samples of all analysed individuals representing all cytotypes of the *Barnardia japonica* complex were preserved in formalin-acetic-acid-alcohol (FAA). Leaf samples were inspected under Nikon SMZ1500 stereomicroscope (Nikon, Japan) and only fully mature leaves were selected for further leaf epidermis analyses (Table 1). Tissue samples were examined using a light microscopy (LM; BX53F, Olympus, Japan) and scanning electron microscopy (SEM; Hitachi E-1010, Japan) following [64]. The micromorphological variation of abaxial (AB) and adaxial (AD) epidermal surfaces of the leaf including epidermal cells and stomatal complexes were analyzed for 13 individuals (at least one individual per cytotype). The guard cells of leaf and pollen grain size were measured from 30 samples per each cytotype (Table S2). Pollen micromorphological characters (i.e., pollen shape and size, exine sculpture) and pollen viability of nine individuals were analyzed using pollen from fertile anthers of flowers collected in the field. Pollen grains were stained using aniline blue dye solution, which only stained fertile pollen grains [67, 68]. Pollen morphological description and terminology followed [69]. Pollen shape and size including the detailed pollen exine ornamentation and the sculpture of apertures were analyzed using SEM following [56, 70].

To test whether the pollen size along the long axis and ploidy level are correlated, Pearson correlation coefficients were computed, and a Mann-Whitney test was conducted to test if the pollen size measurements (long axis) in diploids significantly differ from those in polyploids (GraphPad Prism version 9).

## Results

### Chromosome numbers, karyotypes and genome size variation

**Fig. 2:**
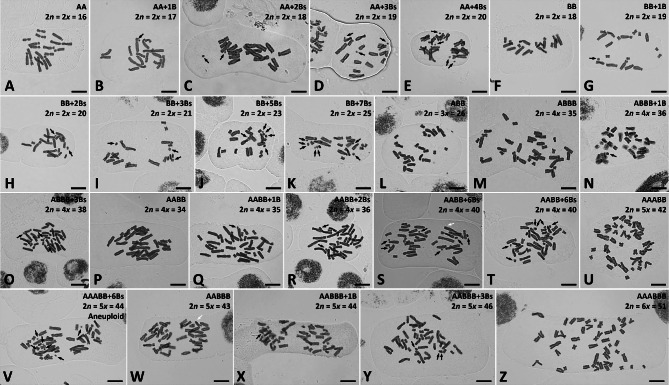
Mitotic metaphase chromosomes of diploid and polyploid cytotypes of the *Barnardia japonica* complex. Black and white arrows indicate B-chromosomes and supernumerary chromosomal segments (SCSs), respectively. Scale bar = 10 μm

Analyses of 131 individuals from 34 populations of the chromosomally hypervariable *Barnardia japonica* complex (Table 1) revealed the presence of both diploid and polyploid individuals. Chromosome numbers in the *B. japonica* complex varied from 2*n* = 16 to 25 in diploids due to the presence or regular A-chromosomes (2*n* = 16 or 2*n* = 18) accompanied by one to seven B-chromosomes and from 2*n* = 26 to 51 in polyploids comprising A-chromosomal sets and one to six Bs as well as two aneuploid individuals (Figs. 1 and 2; Table 1). Five ploidy levels were found including diploids with two distinct base chromosome numbers (*x* = 8 or 9; Fig. 2A, F), triploids (2*n* = 26; Fig. 2L), tetraploids (2*n* = 34, 35; Fig. 2M–T), pentaploids (2*n* = 42, 43; Fig. 2U–Y), and hexaploids (2*n* = 51; Fig. 2Z). Allotetraploid individuals were found most frequently (78 individuals, 59%), whereas allotri- and allohexaploids (one individual each, 1%) were found only sporadically (Figs. 1 and 2). B-chromosomes, easily distinguishable from the A-complement chromosomes by their smaller size (Fig. 2), were frequently found in both diploids (Fig. 2B–E, G–K) and polyploids (Fig. 2N–O, Q–T, V, X–Y) of the *B. japonica* complex (Table 1). Supernumerary chromosomal segments (SCSs), physically integrated into the standard chromosome complement, were occasionally found in polyploids (Fig. 2S, W), whereas aneuploidy was encountered in two individuals (Fig. 2V; Table 1).

**Fig. 3:**
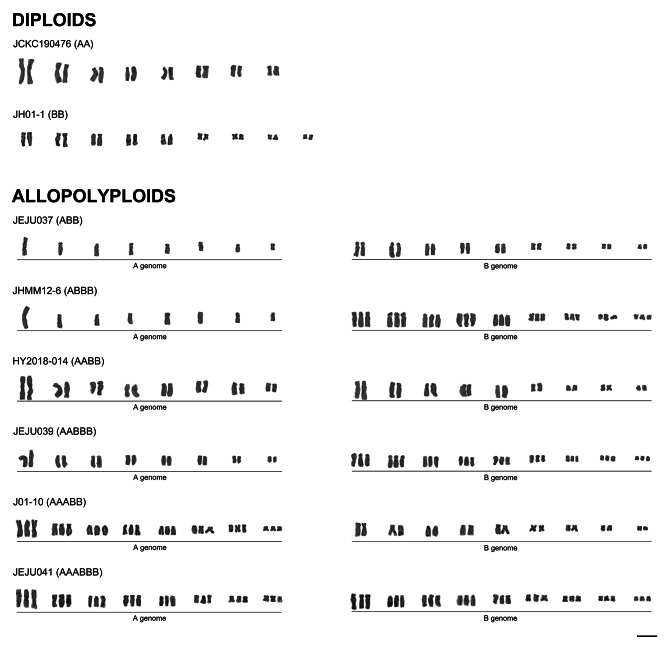
Karyotypes of diploids and polyploids in the *Barnardia japonica* complex. Scale bar = 10 μm

**Fig. 4:**
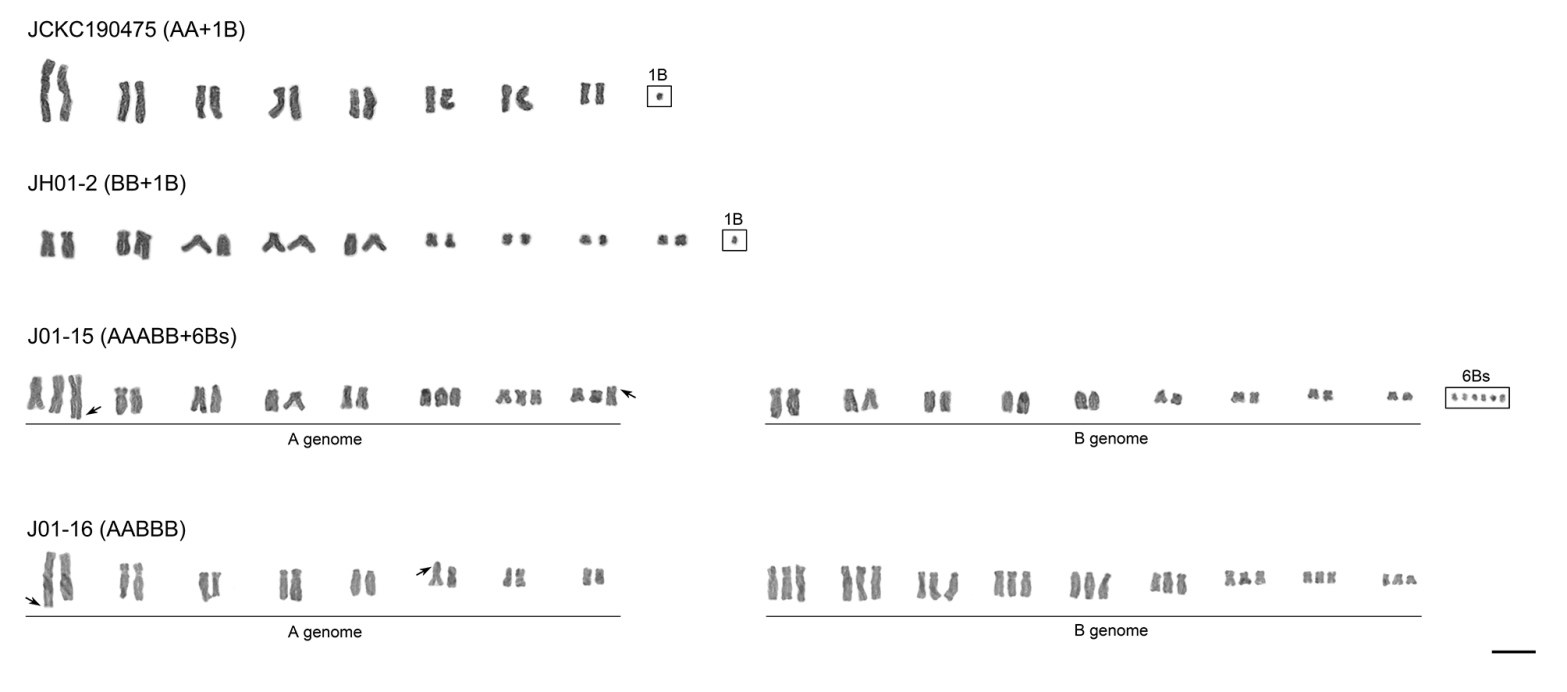
Karyotypes of the selected individuals carrying B-chromosomes (boxes) and supernumerary chromosomal segments (black arrows) in the *Barnardia japonica* complex. Scale bar = 10 μm

Representative karyotypes of all eight cytotypes found among analyzed plants: two diploids (i.e., AA and BB), one triploid (i.e., ABB), two tetraploids (i.e., ABBB and AABB), two pentaploids (i.e., AABBB and AAABB) and one hexaploid (i.e., AAABBB) are presented in Fig. 3. Each polyploid cytotype was easily distinguishable due to different karyotype morphology of the parental diploid cytotypes A and B (Fig. 3). Karyotypes of diploid AA and BB cytotypes were easily distinguishable due to the presence of larger chromosomes in AA (4.00–11.52 μm) compared to BB (2.35–7.45 μm) cytotype (Table S1). The haploid chromosome set of AA cytotype was composed of a large metacentric chromosome (A_1_), the longest subtelocentric chromosome (A_2_), five subtelocentric chromosomes (A_3_–A_7_) and a small metacentric chromosome (A_8_) (Fig. 3). Haploid chromosome set of BB cytotype possessed a subtelocentric chromosome with a satellite at the short arm (B_1_), four subtelocentric chromosomes (B_2_–B_5_) and four small meta- or submetacentric chromosomes (B_6_–B_9_) (Fig. 3). The total haploid chromosome length (HCL) of BB cytotype was lower than that of AA cytotype (42.09 vs. 53.22 μm; Table S1). Karyotype lengths of allopolyploids were not additive compared to their diploid progenitors and ranged from 61.85 μm in allotriploid (expected HKL: ca. 68 μm) to 119.20 μm in allohexaploid (expected HKL: ca. 142 μm; Table S1). Karyotype morphologies of allopolyploids (ABB, ABBB, AABB, AABBB, AAABB, and AAABBB) were additive compared to their diploid progenitors (Fig. 3). Additional genetic materials including both B-chromosomes (one to seven Bs) and supernumerary chromosomal segments (SCSs) have been found in three individuals (Figs. 2 and 4). B-chromosomes were present in both diploids and polyploids. All SCSs were found exclusively in the A subgenome of allopolyploids and were located distally, either within the long or short arms of chromosomes 1, 6, and/or 8 (Figs. 2 and 4).

**Fig. 5:**
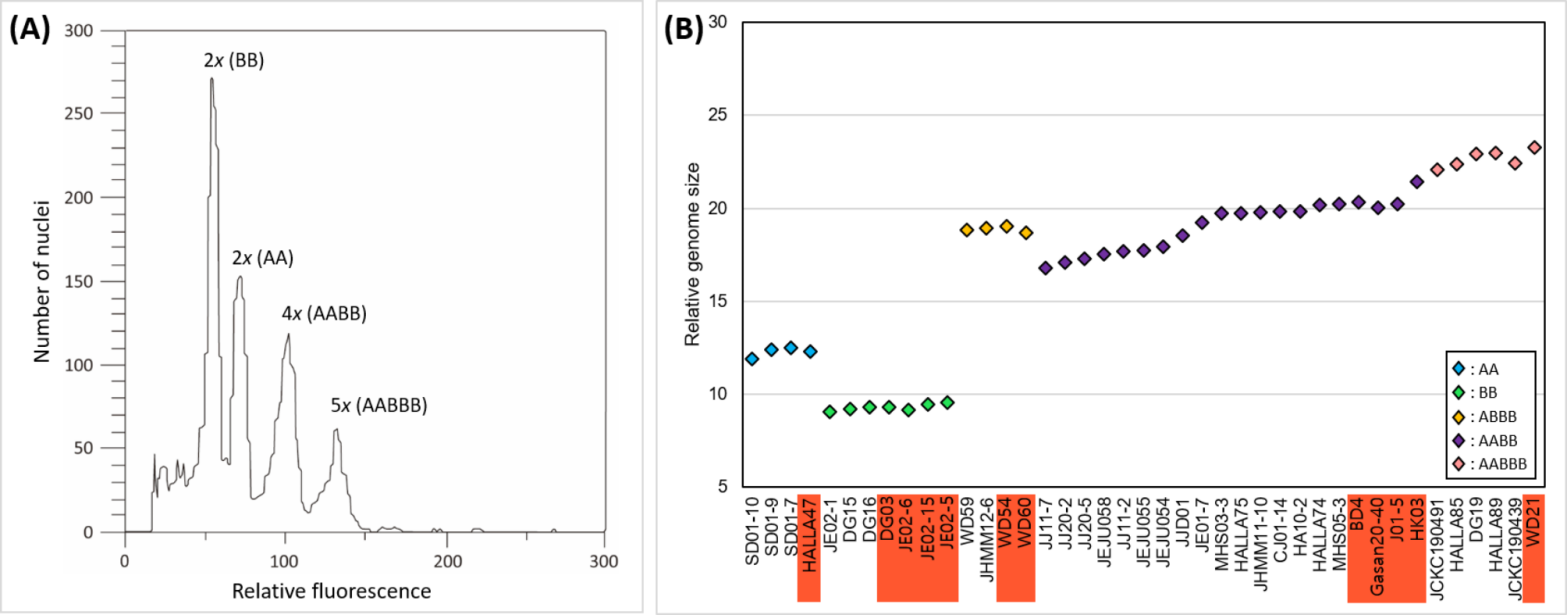
Fluorescence histogram (**A**) and genome size variation (**B**) among analysed *Barnardia japonica* complex individuals (Table 1). Red boxes in (**B**) indicate the individuals carrying B-chromosomes. For collection numbers please refer to Table 1

The DNA amounts (1 C value) of all individuals ranged from 9.05 to 23.23 pg, indicating 2.5-fold difference (Fig. 5A). The coefficient of variation (CV) for the internal standard and sample peaks ranged from 0.92 to 4.49%. The 1 C values were relatively similar with some variation within all cytotypes (Fig. 5B; Table 1). Variation among individuals without B-chromosomes ranged from 9.05 to 22.95 pg and among bulbs with at least one B-chromosome from 9.13 to 23.23 pg (Table 1). Diploid AA cytotype had average genome size of 1 C = 12.23 pg (11.87–12.46 pg), diploid BB cytotype had average 1 C value of 9.17 pg (9.05–9.28 pg), tetraploid ABBB (2*n* = 35) had 1 C of 18.84 pg (18.79–18.90 pg), tetraploid AABB (2*n* = 34) had 1 C of 18.76 pg (17.05–20.26 pg), and pentaploid AABBB (2*n* = 43) had genome size of 22.55 pg (22.03–22.95 pg) (Table 1). The genome sizes of each cytotype with and without B chromosome were not significantly different (AA, *P* = 0.7891; BB, *P* = 0.597; ABBB, *P* = 0.5221; AABBB, *P* = 0.4514) regardless of the number of Bs: AA + 5Bs (12.28 pg), BB + 1B (9.30 pg), BB + 3Bs (9.13–9.52 pg), ABBB + 1B (19.02 pg), ABBB + 2Bs (18.67 pg), AABB + 1B (20.32 pg), AABB + 2Bs (20.03–20.19 pg), AABB + 6Bs (21.38 pg), and AABBB + 3Bs (22.42–23.23 pg), except for AABB and AABB with B chromosomes (*P* < 0.0001) (Fig. 5; Table 1). The 1 C values were cytotype-specific in diploids, but not in polyploids (Fig. 5).

### Leaf and pollen micromorphological characters

**Fig. 6:**
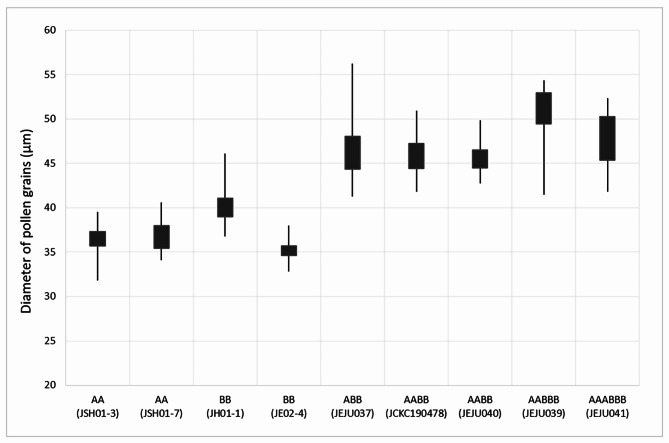
Pollen grain size variation among the diploid and polyploid cytotypes. For collection numbers please refer to Table 1

**Fig. 7:**
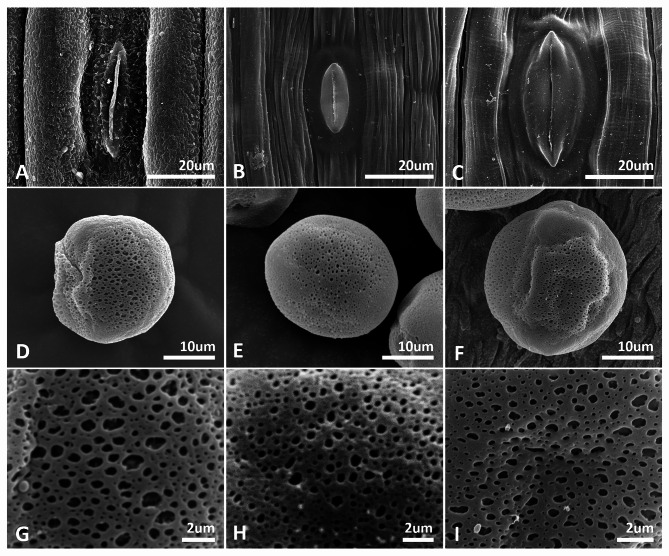
SEM (scanning electron microscope) micrographs of leaf and pollen micromorphological traits of diploids (AA and BB) and AABB allotetraploids of the *Barnardia japonica* complex. (**A**–**C**) Stomatal complex of leaf surface. (**D**–**I**) Pollen grains and exine ornamentation. (**A**, **D**, **G**) AA cytotype, (**B**, **E**, **H**) BB cytotype, (**C**, **F**, **I**) AABB cytotype

The pollen morphological characters including viability, size, apertures and exine ornamentation have been reported herein for the first time for the different ploidy levels (i.e., 2*x*, 3*x*, 4*x*, 5*x* and 6*x*) of *B. japonica* (Figs. 6 and 7, Fig. S2). Pollen grains of all cytotypes were fertile, although sterile pollen grains were frequently found at odd-ploidy levels (ABB and AABBB). The pollen size ranged from 35.13 × 25.98 μm (long × short axis) in diploid BB to 50.33 × 31.99 μm in pentaploid AABBB indicating nearly 1.5-fold difference (Fig. 6). The pollen size and ploidy levels were positively correlated (*r* = 0.78, *P* < 0.0001). Regardless of pollen size variation among the all cytotypes, the exine ornamentations were all reticulate-perforate, and perforations were the major ornamentation in sulcus margin (Fig. 7D–I). The pollen size was significantly different between diploids and polyploids (*P* < 0.0001). The pollen apertures of all cytotypes were consistently monosulcate with granular membranes possessing tiny perforations (Fig. S2).

Sixteen individuals were selected for detailed analyses of leaf surface characters using SEM: four AA diploids, one BB diploid, one ABB triploid, three allotetraploids AABB, two pentaploids (one of each: AABBB and AAABB) and one AAABBB hexaploid, as well as four plants carrying B-chromosomes (Fig. 7A–C; Table 1). The leaf surface of both diploids and polyploids of the *Barnardia japonica* complex lacked trichomes (Fig. S1). AA diploids possessed corrugated cuticle (Fig. 7A, Fig. S1) whereas both BB diploid (Fig. 7B) and AABB allotetraploid (Fig. 7C) had slightly ribbed thickenings of leaf epidermis (Fig. S1). Slightly sunken anomocytic stomata were observed in all the cytotypes and no significant guard cell size variation between abaxial and adaxial surface of the leaves was found in all investigated bulbs of the *B. japonica* complex (Table S2).

## Discussion

### High levels of karyotype and genome size diversity in the *Barnardia japonica* complex

The current study reports for the first time comprehensive analysis of chromosome numbers and genome size variation of all hitherto known cytotypes in the perennial bulbous Korean *B. japonica* complex. The results of the extensive chromosomal survey were largely in agreement with previous reports from Chinese, Japanese, and Korean populations [24, 25, 71]. Genome sizes reported here, however, deviated from those reported by Shibata et al. [22]. The discrepancies (i.e., quite consistent and approximately 2 pg/1 C deviation) are likely to represent methodological problems, as the previous genome size values have been obtained via flow cytometry with less accurate staining of the DNA with DAPI that binds preferentially to AT-rich regions [72] as well as using *Allium fistulosum* as an internal standard [73]. It has been shown that DAPI staining may lead to larger errors in nuclear DNA content evaluation [62], and therefore propidium iodide is used as a standard dye for reliable estimation of genome size in plants using flow cytometry [74–76].

In contrast to the gross morphological uniformity [77], the *B. japonica* complex exhibits remarkable chromosome number and ploidy level variations with occasionally found mixed-ploidy populations, as also reported earlier [25]. Despite the considerable range of chromosome numbers and genome sizes observed in the *B. japonica* complex, this variation is well structured. AA diploids have consistently larger genome sizes than BB diploids (*P* < 0.0001) and there is a positive correlation between genome sizes and ploidy levels. The genome sizes measured in the current study have also provided evidence of genome downsizing in allopolyploids on all ploidy levels (3*x*, 4*x*, 5*x*, and 6*x*), as frequently reported in other plant groups [78–80]. The changes in genome size may be caused by the independent accumulation or reduction of repetitive DNA amounts (e.g., satellite DNAs and/or transposable elements), which contribute to both genome size increases and genome downsizing, which seems to be a general trend in polyploids [81–83]. The repetitive DNA composition of genomes in polyploids has often been shown to be fast-evolving compared to their diploid progenitors. This is often related to the process of genome diploidization [82]. Diploidization processes transform the polyploid genomes back into functional diploids through chromosomal rearrangements, genome downsizing, and gene loss or silencing [84–86]. There is not experimental evidence so far of the processes involved in the genome diploidization in the polyploids of the *B. japonica* complex. However, numerical and structural changes of chromosomes, including genome downsizing, are well studied in allopolyploid genomes as shown in *Brassica* [10], *Melampodium* [11, 12], *Nicotiana* [13], *Prospero* [14], or *Tragopogon* [15]. Further studies employing more sensitive techniques, including molecular phylogenetic analysis using both cpDNA and nrDNA sequences at the populational level, FISH (fluorescence in situ hybridization), and comparative analyses of the repetitive DNA fraction of the genome using next-generation sequencing (NGS) data, will allow for more in-depth analyses of the dynamics of the genome accompanying polyploidization. This will also allow us to identify major repeat types responsible for creating these levels of observed variation.

The occurrence of B-chromosomes reported in the current study is in agreement with earlier studies of the complex from China [24], Japan [71], and Korea [25]. The occurrence of supernumerary genetic material (either B-chromosomes and/or SCS) has frequently been reported not only for *Barnardia*, but also for many other species of the family Hyacinthaceae [41, 42, 87, 88]. Although B-chromosomes have often been reported to contribute to significant variation of genome size [45, 89], no positive correlation between the presence of supernumerary genetic materials and genome size variation has been inferred here for *Barnardia japonica*. This is likely due to rather large absolute genome sizes of *Barnardia japonica* [45]. Similarly, several other studies have also found no significant impact of B-chromosomes or aneuploidy on genome size variation among individuals with larger genome sizes [90–93].

### The utility of micromorphological characters for taxonomical context

Species delimitation is rather complicated and problematic in some genera of the Hyacinthaceae family [14, 27, 60, 94, 95]. This is particularly true for the genera in which rather uniform morphology contrasts with a striking chromosomal variation, caused by dysploidy, polyploidy as well as presence of supernumerary DNA material [96]. Although micromorphological characters including leaf indumentum, reproductive organs, and pollen grains are often useful for species identification in taxonomically complex groups [56–58, 70, 97, 98], no diagnostic structured micromorphological variation has been found in the *B. japonica* complex with exception of some quantitative morphological characters (e.g., leaf diameter, guard cell size, and pollen grain size). Whereas the leaves of BB diploids tend to be narrower with fewer veins when compared to wider leaves with more veins of cytotype AA, these quantitative characters lose their significance once polyploids are included in the comparison. The variation of vegetative morphological characters has been suggested to be associated with genome size variation/polyploidy [99, 100] or with various environmental factors, most likely the availability of the nutrients and water [101], or combination of both. A correlation between ploidy levels and the size of guard cells have been extensively studied [102–105] and has been shown to be related to both genome size changes and to environmental stimuli at the same time, thus being very plastic and not allowing direct correlations of stomatal cell sizes and polyploidy [106–109].

Although we have not analysed populations from the entire range of distribution in the *B. japonica* complex, which extends to China and Japan [22, 24, 26, 28, 35, 59], our data suggest that the complex in Eastern Asia splits into several distinct evolutionary units on the basis of the available cytological and micromorphological evidence. Further studies involving molecular phylogenomic and cytogenetic analysis of populations representing whole distribution range of the complex, however, are still required to understand the genetic variation as well as the evolutionary history of the complex and test the existing taxonomic treatments.

Odd-ploidy level polyploids (3*x*, 5*x*, 7*x*, 9*x*, etc.) generally face difficulties in the production of functional gametes owing to irregularities in meiosis and are, therefore, expected to produce high-sterile pollen grains and seeds [110, 111]. On the other hand, triploid may also act as triploid bridge relevant for recurrent origin of higher (and even) ploidy levels [8]. Similarly to other various species complex groups [8], the odd-ploidy cytoytpes occurred in low frequency in the *B. japonica* complex, and pollen sterility in triploids was higher than that of pentaploids, as reported also in the *Senecio carniolicus* complex [112]. Although odd-ploidy individuals have been considered an instant postzygotic barrier due to the uneven number of chromosome sets caused by irregularities during meiosis [113, 114], the barrier is often leaky and gene flow may be mediated by at least partially fertile interploidy hybrids [8, 110–112, 115].

Pollen micromorphological analyses provided some evidence of correlation of ploidy levels and pollen sizes, with diploid taxa having significantly smaller pollen grains compared to all polyploid cytotypes, as reported also for other plant groups [116–118]. These differences were, however, not very pronounced. Although pollen exine ornamentations have been shown to be of great taxonomic significance in Hyacinthaceae [50, 95, 119, 120], the exine sculpturing in *Barnardia japonica* has shown no variation with reticulate-perforate sculpturing in all analyzed cytotypes. Thus, none of the analyzed leaf and pollen micromorphological characters were of any taxonomic significance and thus cannot be used for identification of the cytotypes in the *B. japonica* complex. This strongly contrasts with very structured and established karyotypical variation of the group. Clearly, chromosomal changes are one of the driving forces of the evolution of this species complex. Further population genetic and cytogenomic analyses of all ploidy levels from whole distribution area of the species should provide more information about the role of chromosomal changes in the diversification within this complex [121–124].

## Conclusions

The present study constitutes the first step towards a better understanding of the evolutionary history of the *Barnardia japonica* complex. Although no structured diagnostic micromorphological variation has been found in the *B. japonica* complex, the reported structured variation of chromosome numbers and genome sizes, provides a baseline for further molecular cytogenetic analyses. Genome skimming NGS data will allow for characterization of complete repeat profiles of all cytotypes and will guide the selection of various repeat types that can be used as chromosomal markers. Further molecular phylogenetic analyses of the FISH-karyotyped individuals will allow testing the existing hypotheses on taxonomic treatments of this species complex and will eventually allow to resolve the taxonomic status of the cytotypes of the *B. japonica* complex.

### Electronic supplementary material

Below is the link to the electronic supplementary material.

Supplementary Material 1

## Data Availability

All relevant data are within the paper and its Supporting Information files. The data presented in this study will be available on request from the corresponding authors.
