# Supplementary material for: Does the evolution of micromorphology accompany chromosomal changes on dysploid and polyploid levels in the Barnardia japonica complex (Hyacinthaceae)?
Source: BMC Plant Biol. 2023 Oct 11;23:485. doi: 10.1186/s12870-023-04456-9 (PMC10565974; doi:10.1186/s12870-023-04456-9)

# Does the evolution of micromorphology accompany chromosomal changes on dysploid and polyploid levels in the *Barnardia japonica* complex (Hyacinthaceae)?

Hyeonjin Kim^1†^, Bokyung Choi^1†^, Changyoung Lee^2^, Jin-Hyub Paik^2^, Chang-Gee Jang^3^, Hanna Weiss-Schneeweiss^4*^, Tae-Soo Jang^1*^

^1^Department of Biological Science, College of Bioscience and Biotechnology, Chungnam National University, Daejeon, Republic of Korea

^2^International Biological Material Research Center, Korea Research Institute of Bioscience and Biotechnology, Daejeon, Republic of Korea

^3^ Department of Biology Education, Kongju National University, Gongju, 32588, Republic of Korea

^4^Department of Botany and Biodiversity Research, University of Vienna, A-1030 Vienna, Austria

*** Correspondence:**Hanna Weiss-Schneeweiss
hanna.schneeweiss@univie.ac.at

Tae-Soo Jang
jangts@cnu.ac.kr

**^†^ These authors contributed equally to this work**

**Supplementary Table 1.**  Karyotype statistics of analyses of diploids and polyploids in the *Barnardia japonica* complex.

| Cytotype (Coll. No.) | Chromosome length (μm) | | | AsI (%) | RI |
| --- | --- | --- | --- | --- | --- |
|  | Largest | Smallest | HKL |  |  |
|  | Mean ± S.D. | Mean ± S.D. | Mean ± S.D. |  |  |
| AA (SD01-7) | 11.67 ± 0.83 | 4.26 ± 0.40 | 54.62 ± 3.97 | 67.40% | 2.74 |
| AA (JCKC190476) | 11.52 ± 1.88 | 4.00 ± 0.81 | 53.22 ± 7.64 | 70.70% | 2.81 |
| AA+1B (JCKC190475) | 13.52 ± 0.63 | 4.62 ± 0.26 | 64.57 ± 1.64 | 74.35% | 2.93 |
| AA + 2Bs (HH01-6) | 10.62 ± 0.79 | 3.92 ± 0.33 | 50.17 ± 1.78 | 66.53% | 2.72 |
| BB (JH01-1) | 7.45 ± 0.55 | 2.35 ± 0.25 | 42.09 ± 3.82 | 73.30% | 3.17 |
| BB (JE02-4)* | 8.03 | 2.14 | 46.77 | 74.89% | 3.72 |
| BB + 1B (JH01-2)* | 6.46 | 2.05 | 38.53 | 73.12% | 3.16 |
| ABB (JEJU037) | 9.70 ± 2.27 | 2.20 ± 0.10 | 61.85 ± 4.48 | 72.50% | 4.40 |
| ABBB (JHMM12-6) | 12.66 ± 1.33 | 2.59 ± 0.24 | 101.34 ± 8.32 | 71.90% | 4.88 |
| AABB (J01-9) | 12.16 ± 2.35 | 2.44 ± 0.14 | 98.72 ± 15.95 | 72.77% | 4.98 |
| AABB (HY2018-014) | 10.46 ± 1.45 | 2.26 ± 0.26 | 90.20 ± 10.71 | 69.70% | 4.62 |
| AABB (C01-1)* | 11.82 | 2.83 | 98.60 | 69.19% | 4.17 |
| AABB (JEJU040)* | 8.62 | 1.86 | 75.89 | 73.31% | 4.62 |
| AABB + 1B (JCKC190479)* | 10.89 | 2.26 | 93.22 | 72.70% | 4.82 |
| AABB + 5Bs (J01-13) | 11.02 ± 1.33 | 3.05 ± 0.35 | 108.02 ± 12.89 | 71.37% | 3.61 |
| AABB + 6Bs (KHJ05) | 13.42 ± 0.72 | 2.74 ± 0.53 | 126.49 ± 6.05 | 70.81% | 4.90 |
| AABBB (J01-16) | 11.85 ± 1.53 | 2.26 ± 0.80 | 119.23 ± 20.33 | 72.22% | 5.24 |
| AABBB (J01-17)* | 10.84 | 1.73 | 102.21 | 72.34% | 6.26 |
| AABBB (JEJU039) | 9.05 ± 0.65 | 2.22 ± 0.29 | 99.37 ± 9.72 | 72.70% | 4.07 |
| AAABB (J01-10) | 8.32 ± 0.55 | 1.76 ± 0.03 | 92.66 ± 6.04 | 70.60% | 4.74 |
| AAABB + 6Bs (J01-15) | 10.46 ± 0.71 | 2.37 ± 0.43 | 101.57 ± 9.83 | 71.79% | 4.41 |
| AAABBB (JEJU041) | 9.45 ± 0.40 | 2.47 ± 0.12 | 119.20 ± 3.14 | 71.20% | 3.83 |

Note: AsI (asymmetry index): the ratio of all long arms to the total haploid chromosome length; RI (ratio index): the ratio of the longest to the shortest chromosome; HKL: total haploid chromosome length with standard deviation (S.D.). Asterisks indicate individuals for which only one chromosomal spread was used for measurements.

**Supplementary Table 2. Guard cell size variation among the cytotypes in the *Barnardia japonica* complex.**

| Cytotype (Coll. No.) | 2*n* | Length of guard cell (μm) | |
| --- | --- | --- | --- |
|  |  | Abaxial (Mean ± S.D.) | Adaxial (Mean ± S.D.) |
| AA (JCKC190422) | 16 | 37.96 ± 2.09 | 27.42 ± 1.98 |
| AA (JCKC190476) | 16 | 31.59 ± 1.69 | 29.96 ± 2.76 |
| AA (JSH01-1) | 16 | 41.98 ± 3.98 | 35.03 ± 2.04 |
| AA+2Bs (HH01-6) | 18 | 30.88 ± 2.43 | 25.67 ± 1.50 |
| BB (JH01-3) | 18 | 22.97 ± 2.01 | 18.47 ± 1.71 |
| BB+1B (JH01-2) | 19 | 26.11 ± 1.56 | 24.10 ± 1.81 |
| ABB (JEJU037) | 26 | 35.57 ± 2.51 | 30.00 ± 1.83 |
| ABBB+3Bs (HY2018-003) | 38 | 35.82 ± 3.01 | 30.88 ± 2.21 |
| AABB (HY2018-008) | 34 | 30.42 ± 3.40 | 28.54 ± 2.46 |
| AABB (JEJU040) | 34 | 29.28 ± 3.04 | 24.58 ± 2.08 |
| AABBB (J01-17) | 43 | 33.60 ± 3.17 | 30.28 ± 1.70 |
| AAABB (J01-10) | 42 | 35.17 ± 2.11 | 28.88 ± 3.45 |
| AAABBB (JEJU041) | 51 | 36.54 ± 3.17 | 24.21 ± 1.34 |

**Supplementary Table 3. Pollen grains size variation among the representative cytotypes of the *Barnardia japonica* complex.**

| Cytotype (Coll. No.) | Pollen grain size (μm) | | Exine thickness (μm) | |
| --- | --- | --- | --- | --- |
|  | Long axis  (Mean ± S.D.) | Short axis  (Mean ± S.D.) | Proximal  (Mean ± S.D.) | Near aperture  (Mean ± S.D.) |
| AA (JSH01-3) | 36.31 ± 1.95 | 30.31 ± 3.43 | 1.43 ± 0.30 | 1.16 ± 0.27 |
| AA (JSH01-7) | 36.83 ± 1.90 | 32.41 ± 3.13 | 0.92 ± 0.15 | 0.80 ± 0.17 |
| BB (JH01-1) | 40.37 ± 2.08 | 25.92 ± 1.34 | 1.02 ± 0.25 | 0.87 ± 0.12 |
| BB (JE02-4) | 35.13 ± 1.21 | 25.98 ± 1.93 | 1.03 ± 0.15 | 0.83 ± 0.10 |
| ABB (JEJU037) | 46.54 ± 3.56 | 32.83 ± 2.76 | 1.06 ± 0.18 | 0.88 ± 0.15 |
| AABB (JCKC190478) | 47.78 ± 2.50 | 33.71 ± 1.97 | 1.04 ± 0.19 | 0.84 ± 0.21 |
| AABB (JEJU040) | 45.59 ± 1.72 | 32.56 ± 2.23 | 1.18 ± 0.30 | 0.96 ± 0.21 |
| AABBB (JEJU039) | 50.33 ± 3.67 | 31.99 ± 2.99 | 1.26 ± 0.21 | 0.98 ± 0.19 |
| AAABBB (JEJU041) | 47.94 ± 3.23 | 30.18 ± 2.65 | 1.14 ± 0.16 | 0.93 ± 0.18 |

**Supplementary Figure 1.** SEM (scanning electron microscope) micrographs of leaf micromorphological characters of diploid (AA and BB) and allopolyploid cytotypes in the *Barnardia japonica* complex. **(A)** AA (JCKC190422), **(B)** AA (JCKC190476), **(C)** AA (JSH01-1), **(D)** BB (JH01-3), **(E)** BB+1B (JH01-2), **(F)** ABB (JEJU037), **(G)** ABBB+3Bs (HY2018-003), **(H)** AABB (HY2018-007), **(I)** AABB (HY2018-008), **(J)** AABB (JEJU040), **(K)** AABB (J01-17), **(L)** AABBB (J01-10), **(M)** AAABB+6Bs (J01-11), **(N)** AAABBB (JEJU041).


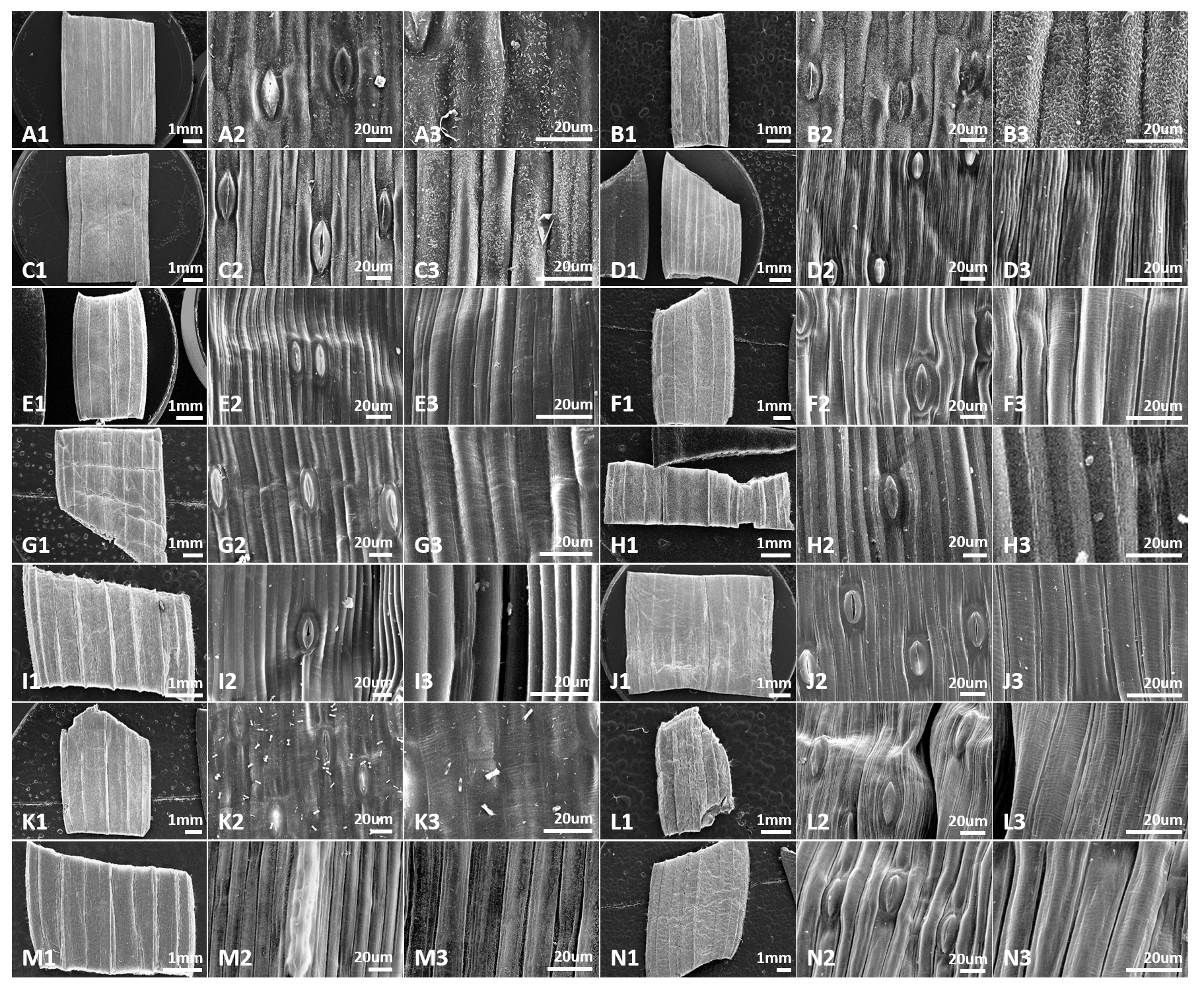


**Supplementary Figure 2.** Fertile pollen grains of the diploid and polyploid cytotypes of the *Barnardia japonica* complex stained with aniline blue dye solution. Scale bars = 20 μm.


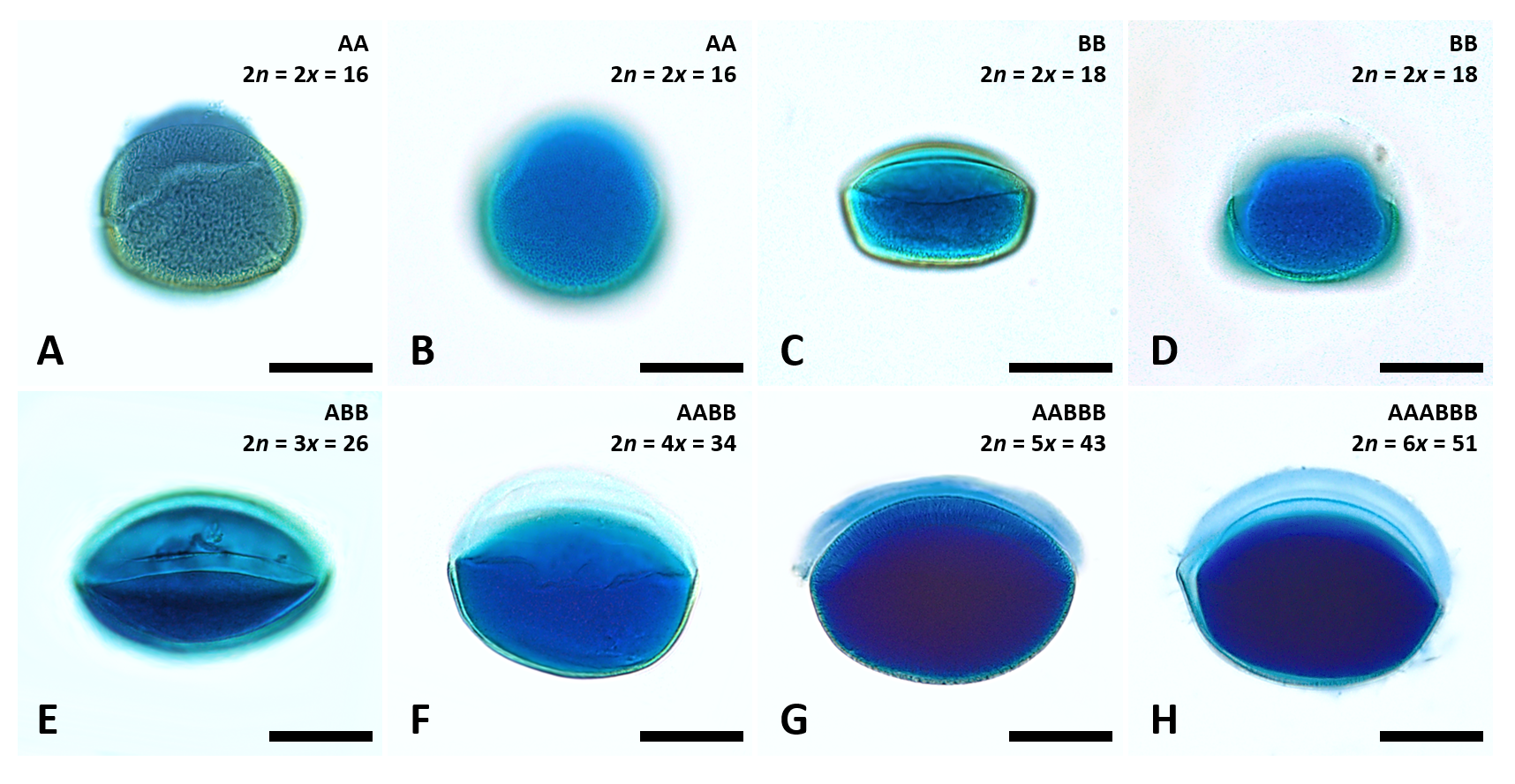

Supplement: Supplementary file 1 — Supplementary Material 1 [file 12870_2023_4456_MOESM1_ESM.doc]
